# Semi-infectious particles contribute substantially to influenza virus within-host dynamics when infection is dominated by spatial structure

**DOI:** 10.1093/ve/vead020

**Published:** 2023-03-21

**Authors:** Alex Farrell, Tin Phan, Christopher B Brooke, Katia Koelle, Ruian Ke

**Affiliations:** Department of Mathematics, University of Arizona, Tucson, AZ 85721, USA; T-6, Theoretical Biology and Biophysics, Los Alamos, NM 87545, USA; Department of Microbiology, University of Illinois at Urbana-Champaign, Urbana, IL 61801, USA; Department of Biology, Emory University, Atlanta, GA 30322, USA; T-6, Theoretical Biology and Biophysics, Los Alamos, NM 87545, USA; Department of Microbiology, University of Illinois at Urbana-Champaign, Urbana, IL 61801, USA

**Keywords:** influenza virus, within-host dynamics, semi-infectious particle, coinfection, spatial structure

## Abstract

Influenza is an ribonucleic acid virus with a genome that comprises eight segments. Experiments show that the vast majority of virions fail to express one or more gene segments and thus cannot cause a productive infection on their own. These particles, called semi-infectious particles (SIPs), can induce virion production through complementation when multiple SIPs are present in an infected cell. Previous within-host influenza models did not explicitly consider SIPs and largely ignore the potential effects of coinfection during virus infection. Here, we constructed and analyzed two distinct models explicitly keeping track of SIPs and coinfection: one without spatial structure and the other implicitly considering spatial structure. While the model without spatial structure fails to reproduce key aspects of within-host influenza virus dynamics, we found that the model implicitly considering the spatial structure of the infection process makes predictions that are consistent with biological observations, highlighting the crucial role that spatial structure plays during an influenza infection. This model predicts two phases of viral growth prior to the viral peak: a first phase driven by fully infectious particles at the initiation of infection followed by a second phase largely driven by coinfections of fully infectious particles and SIPs. Fitting this model to two sets of data, we show that SIPs can contribute substantially to viral load during infection. Overall, the model provides a new interpretation of the *in vivo* exponential viral growth observed in experiments and a mechanistic explanation for why the production of large numbers of SIPs does not strongly impede viral growth. Being simple and predictive, our model framework serves as a useful tool to understand coinfection dynamics in spatially structured acute viral infections.

## Introduction

Influenza A viruses (IAVs) cause hundreds of thousands of hospitalizations and tens of thousands of deaths and cost tens of billions of dollars each year in the USA alone (Molinari et al. 2007). In addition, pandemic strains periodically emerge from the reassortment of human, swine, and/or bird strains, leading to high morbidity and mortality rates (Taubenberger and Morens 2006). Extensive efforts have been made to understand the transmission and evolution of IAVs at the epidemiological level (Nsoesie et al. 2014); however, our understanding of the molecular origins of the within-host genomic diversity of IAVs and how this diversity affects infection dynamics is incomplete (Brooke 2017). This knowledge may be crucial for understanding the within-host IAV evolution and the frequency of *in vivo* reassortment and ultimately for the development of effective vaccines and treatment strategies (Brooke 2014; 2017).

IAVs are negative-sense ribonucleic acid (RNA) viruses with genomes that comprise eight gene segments. Proteins from all eight segments are essential for the completion of the viral replication cycle. The majority of virions (between 70 per cent and 98 per cent) fail to express a complete set of genome segments and thus are non-infectious under traditional limiting dilution assays (Brooke et al. 2013). These non-infectious virions are broadly categorized as ‘semi-infectious particles’ (SIPs), whereas, in contrast, virions capable of delivering functional copies of all eight segments are termed ‘fully infectious particles’ (FIPs). If two SIPs infect the same cell, however, they can complement each other to express at least one copy of all eight gene segments and thereby be capable of producing viral progeny that will go on to infect other cells (Hirst and Pons 1973; Brooke 2014a). This phenomenon, called ‘multiplicity reactivation’ or ‘complementation’, provides SIPs the potential to induce a productive infection within a cell in the absence of FIPs. Importantly, the presence of SIPs has been shown to enhance IAV reassortment and diversity and thus may accelerate the rate of IAV evolution (Fonville et al. 2015). Despite its importance, understanding of the extent to which the coinfection of SIPs occurs and contributes to overall viral dynamics is lacking.

**Figure 1.**
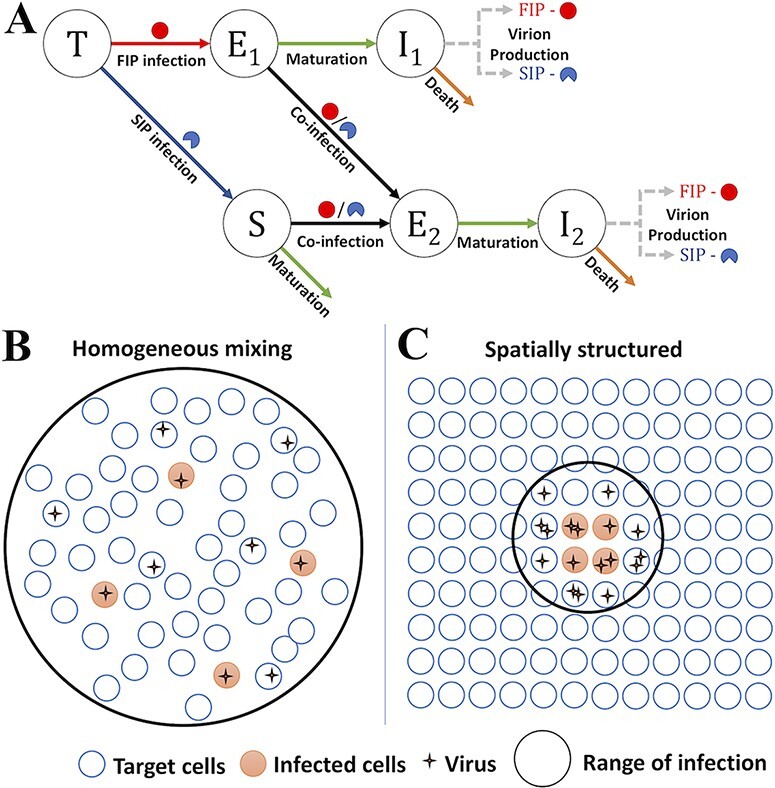
The model schematics and assumptions about the infection processes in the two distinct models analyzed in the study. (A) A schematic of the within-host influenza model that includes SIPs and FIPs, respectively. FIPs are represented by full circles and SIPs by semicircles. Uninfected target cells (*T*) can be infected by FIPs and SIPs to become cells in an eclipse phase (*E_1_* and *S*, respectively). When cells in an eclipse phase (*E_1_* or *S*) are further infected by FIPs or SIPs, they become coinfected cells in a different eclipse phase *E_2_. E_1_* and *E_2_* cells mature into productively infected cells (*I_1_* and *I_2_*, respectively) to produce FIPs and SIPs. (B) The assumptions present in the MA model. When cells and virions are homogeneously mixed, a virus encounters all target cells at an equal probability. As a result, coinfection will be extremely rare when most target cells are uninfected. (C) The assumptions present in the TS model. When the infection spreads in a spatially structured manner, a virus can only reach a limited number of neighboring cells. As a result, when many viruses are produced within a local area, coinfection can occur frequently.

Previously, mathematical models have been used to describe IAV viral load dynamics (Baccam et al. 2006), estimate drug efficacies (Beauchemin et al. 2008; Canini et al. 2014), and characterize the role of innate and adaptive immunity (Miao et al. 2010; Saenz et al. 2010; Pawelek et al. 2012). While some models have included ‘non-infectious’ virions (Schulze-Horsel et al. 2009; Pinilla et al. 2012; Paradis et al. 2015), the roles that coinfection and complementation between SIPs play in regulating within-host IAV dynamics have not been studied. This knowledge is crucial to a quantitative understanding of the extent of coinfection and reassortment during infection (Fonville et al. 2015). To address this gap, we constructed two distinct viral dynamic models that keep track of the dynamics of SIPs and FIPs during an infection (see Fig. 1 for a schematic). The two models differ in their assumptions of the infection process. In the first model, the infection of target cells by a virus is assumed to be homogeneously mixed (Fig. 1B), whereas in the second model, we implicitly consider the spatial structure of host cells and assume that a virus can only infect a fraction of target cells (Fig. 1C). We show that considering the impact of spatial structure on the availability of target cells is critical to reproduce key features of published experimental data. We further fit the model with the implicit spatial structure to *in vivo* data and evaluate the role of SIPs and coinfection in driving IAV dynamics.

## Results

### The mass-action model

We first constructed a model including SIP and FIP coinfection dynamics assuming homogeneous mixing between viral particles and cells (Fig. 1B), an assumption commonly made in viral dynamic models (Baccam et al. 2006; Pawelek et al. 2012; Smith and Perelson 2011). We term this model the mass-action (MA) model because MA terms are used to model the infection process under homogeneous mixing. It considers target cells (*T*) for infection, FIPs (*V_F_*), SIPs (*V_S_*), cells singly infected by an FIP (*E_1_* and *I_1_*), or an SIP (*S*) and coinfected by FIPs and/or SIPs (*E_2_* and *I_2_*). See Methods for the ordinary differential equations (ODEs) and detailed descriptions of the model. This model is an extension of a viral dynamic model that assumes target cell limitation (Baccam et al. 2006), and it ignores the role of the innate and adaptive immune responses to IAV infection. While previous modeling work showed that the adaptive immune responses were necessary to explain the clearance of the virus following peak viremia (Pawelek et al. 2012), we here adopt the simpler target cell–limited model structure because we primarily aim to use our models to understand the coinfection dynamics during exponential virus growth that occurs prior to peak viremia.

### Predictions of the MA model are inconsistent with observations of frequent coinfection of IAV

**Figure 2.**
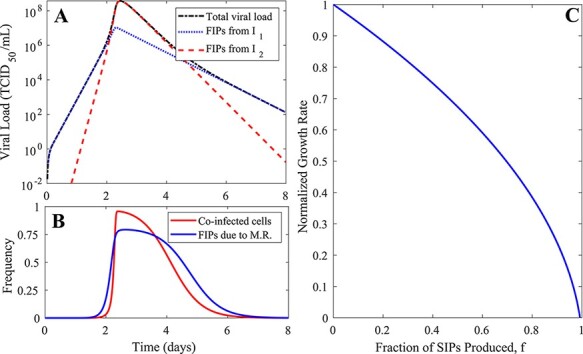
The simulation results and predictions of the MA model. (A) The viral load dynamics from a simulation of the MA model. The total viral load is shown, alongside partitions of viral load that result from singly and coinfected cells (*I*_1_ and *I*_2_, respectively). Measured using TCID_50_, the total viral load is the concentration of FIPs that stem from all sources. FIPs from *I_1_* and *I_2_* are the concentrations of fully infectious viruses that stem from singly and coinfected virion-producing cells, respectively. See the ‘Parameter values, simulation, and data-fitting procedure’ section in Methods for the parameter values used to generate the simulation results. (B) The proportion of infected cells that are coinfected and the proportion of fully infectious virions that originate as a consequence of multiplicity reactivation (M.R.). (C) The exponential viral growth rate is strongly dependent on the fraction of SIPs produced (*f*). The viral growth rate here is normalized relative to the growth rate when no SIPs are produced.

We first used the MA model to gauge the relative contribution of singly infected cells, coinfected cells, and multiplicity reactivation (due to complementation of SIPs) to viral load dynamics during the exponential growth. The MA model predicts that the viral population grows in two phases. The first phase lasts for the majority of the viral growth period, whereas the second phase lasts only for a short period before the viral peak. The two phases are driven by the FIPs produced from singly infected and coinfected cells, respectively (Fig. 2A). Extending the model to explicitly keep track of FIPs produced through complementation (Fig. S1), we found that complementation does not significantly contribute to the first phase of viral growth and coinfection is infrequent until a large fraction of cells become infected during the second phase (Fig. 2B). These predictions arise from the homogeneous mixing assumption and are largely insensitive to the specific parameter values assumed. This is because during the large proportion of the viral growth period, i.e. the first viral growth phase, the number of uninfected cells is orders of magnitude higher than infected cells. Under the assumption of homogeneous mixing, a virus will most likely encounter and infect an uninfected cell (Fig. 1B). The infection of already infected cells occurs at extremely low frequencies until the viral load increases close to the peak where the number of already infected cells exceeds the number of uninfected cells. This prediction, however, is inconsistent with the experimental data suggesting that coinfection is frequent throughout the course of infection (Marshall et al. 2013; Brooke 2014; Fukuyama et al. 2015).

We then asked how the first viral growth phase is affected by the fraction of viral progenies that are assumed to be SIPs, under the constraint that the total number of viral progenies produced from infected cells remains the same. We first derived an analytical approximation to the rate of exponential growth (denoted by ${\lambda _{MA}}$) (see S1 Supporting Information):


(1) $${\lambda _{MA}} \approx \frac{1}{2}\left( { - {\delta _1} - k + \sqrt {{{\left( {{\delta _1}{\rm{\, + \,k}}} \right)}^2} + 4k{\delta _1}\left( {{\rm{\,}}{R_0}\left( {1 - f} \right) - 1} \right)} } \right),$$

where ${\delta _1}$ and $k$ are the death rate of cells singly infected by FIP (*I_1_*) and the rate of transition from eclipse cells to productively infected cells, respectively, and ${R_0} = \frac{{T\left( 0 \right){\beta _{MA}}{p_1}}}{{c{\delta _1}}}$ (see Methods for definition of parameters). Note that ${R_0}$ here is the average number of cells singly infected by an FIP when a cell singly infected by an FIP is introduced into the system in the case where *f* = 0, i.e. when all virions produced from infected cells are FIPs.

This expression suggests that the rate of exponential growth ${\lambda _{HM}}$ is very sensitive to changes in the fraction of viral progeny that are SIPs. When all particles produced are FIPs, $f = 0$ and the rate of viral growth ${\lambda _{HM}}$ is at its maximum. When all viruses produced from infected cells are SIPs, $f = 1$ and ${\lambda _{HM}} \lt 0$. The MA model, therefore, predicts that viral load would decrease, and an infection would not be able to take off. The simulation of the model confirms these analytical results (Fig. 2C). However, these model predictions are inconsistent with a recent study that showed that a mutant influenza virus population that is unable to produce FIPs (corresponding to $f = 1$) can grow to high viral loads in guinea pigs (Jacobs et al. 2019). Furthermore, among different IAV strains, SIPs compose between 70 per cent and 98 per cent of biologically active particles in an IAV population (Brooke et al. 2013). While the ratio of virions produced that are semi-infectious is not the only difference between different IAV strains, the high fraction and the large variation of SIP production (Brooke et al. 2013, 2014) suggest that IAV growth is not enormously sensitive to variations in the fraction of SIP production. These inconsistencies strongly argue that the MA model is not a good model to describe coinfection or to quantify the extent to which SIPs can contribute to within-host viral dynamics.

### The target cell saturation model

IAV spreads from the upper respiratory tract to the lower respiratory tract, and thus, spatial structure is an inherent property of IAV infection (Gallagher et al. 2018). A key feature of spatial spread is that during infection, a virus cannot reach all target cells in a host; instead, there are only a small number of neighboring target cells that a virus can access (Fig. 1C). To incorporate this feature, we considered an alternative model using Michaelis–Menten (also known as Holling type II) functions to model the availability of susceptible cells (see Methods). We argue that the assumption of target cell saturation (TS) is more biologically plausible, especially for influenza infections in tissue. For example, it has been shown in the human tracheobronchial epithelium that foci of infected cells formed during infection and a virion produced may be more likely to infect cells that are closer to the cells that produced the virion (Matrosovich et al. 2004). Similar results have also been observed in the lungs of mice (Heaton et al. 2013; Pan et al. 2013; Tran et al. 2013) and ferrets (van den Brand et al. 2012).

### The TS model is consistent with observations of within-host infection

We analyzed the TS model and found that as in the MA model, there exist two phases of exponential growth. However, the duration of the first phase is parameter dependent. The viral growth during the first phase is driven by FIP production from singly infected cells because the viral load is low, the number of *E_1_* and *S* cells (which are targets for coinfection) are low, and thus coinfection is infrequent. When the viral load increases to a level (set by the Michaelis–Menten functions in the model) such that the number of *E*_1_ and *S* cells is higher, it becomes likely that a virus infects an already infected cell and coinfection occurs more frequently. The viral load then enters the second phase of exponential growth that is driven by viral production from coinfected cells. During this phase, both FIPs and SIPs contribute to viral growth. The transition from the first to the second growth phase is thus determined by viral load and the Michaelis constant, ${K_M}$, in the Michaelis–Menten function (Fig. S2). Note that we expect the Michaelis constant to be very small because for influenza infection in tissue, the number of target cells that a virus can reach is expected to be small. Consequently, we expect the first growth phase to only last for a very short period of time.

We derived approximations of the rates of the two exponential growth phases as follows (see S1 Supporting Information):


(2) $${\lambda _1} \approx \frac{1}{2}\left( { - {\delta _1} - k + \sqrt {{{\left( {{\delta _1}{\rm{ + \,k}}} \right)}^2} + 4k{\delta _1}\left( {{R_{0,1}}\left( {1 - f} \right) - 1} \right)} } \right),$$

(3) $${\lambda _2} \approx \frac{1}{2}\left( { - {\delta _2} - k + \sqrt {{{\left( {{\delta _2}{\rm{\, + \,k}}} \right)}^2} + 4k{\delta _2}\left( {{R_{0,2}}\left( {\frac{{1 - f}}{2} + \frac{{{p_2}}}{{2{p_1}}}} \right) - 1} \right)} } \right).$$

where ${\delta _1}$, ${\delta _2}$, *k*, and *f* are as defined previously in the MA model. Here, ${p_1}$ and ${p_2}$ are the viral production rates from singly and coinfected cells, respectively, and ${R_{0,1}} = \frac{{{\beta _{TS}}{p_1}}}{{c{\delta _1}}}$ and ${R_{0,2}} = \frac{{{\beta _{TS}}{p_1}}}{{c{\delta _2}}}$ (see Methods for definition of parameters).

**Figure 3.**
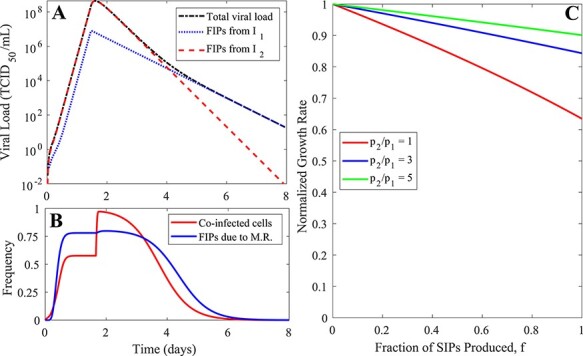
The simulation results and predictions of the TS model. (A) The viral load dynamics and drivers of them predicted by the TS model. See the ‘Parameter values, simulation, and data-fitting procedure’ section in Methods for the parameter values used to generate the simulation results. (B) The frequency of infected cells that are coinfected and the frequency of fully infectious virions that are produced due to multiplicity reactivation (M.R.) of SIPs. (C) The rate of the second viral growth phase is relatively insensitive to the fraction of SIPs produced (*f*), especially when the virus production from coinfected cells is much higher than the virus production from singly infected cells (e.g. *p*_2_/*p*_1_ = 5).

We then tested the extent to which the second growth phase ${\lambda _2}$ depends on the fraction of viral progeny produced that are SIPs (*f*). Our model predicts that when coinfected cells produce more virions than singly infected cells as shown by Martin et al. (2020) (for example, *p*_2_/*p*_1_ = 3 or 5 in Fig. 3C), the growth rate becomes insensitive to changes in the fraction of SIP production. This result stands in stark contrast to the findings of the MA model, where the rate of viral growth declines rapidly with higher fractions of SIP production. Therefore, only the results from our spatial model provide a plausible explanation for how IAV can afford to produce a large fraction of SIPs.

Overall, the TS model makes predictions that are consistent with biological observations, such as frequent coinfections during most of the course of infection (Marshall et al. 2013; Brooke 2014; Fukuyama et al. 2015) and insensitivity to changes in the fraction of SIPs produced (Brooke et al. 2014; Brooke 2017). We thus argue that the TS model is preferred over the MA model and that the spatial structure is an important factor in influenza infection (Gallagher et al. 2018).

### Quantification of coinfection frequency through fitting the TS model to published human and animal datasets

We then fitted the TS model to two sets of data to estimate the frequency of coinfection and the contribution of SIPs toward the viral load. In one study, six unvaccinated ponies were infected with the equine influenza virus (H3N8) (Pawelek et al. 2012). Nasal secretions were collected daily for 10 days postinfection and virus RNA levels were quantified (Quinlivan et al. 2007). In another study, six sero-susceptible human adult participants were experimentally infected with a cloned IAV, followed by daily nasal washes for 1 week to measure the patients’ viral titers (Murphy et al. 1980; Baccam et al. 2006). We took a nonlinear mixed-effect modeling approach to fit the TS model to data from all individuals simultaneously (Methods). We first tested if a covariant for the different datasets is needed (i.e. testing if the population estimates differ between the two datasets) and found that a model without a covariant is the best model to describe the datasets according to the corrected Bayesian information criterion (Table S1).

**Figure 4.**
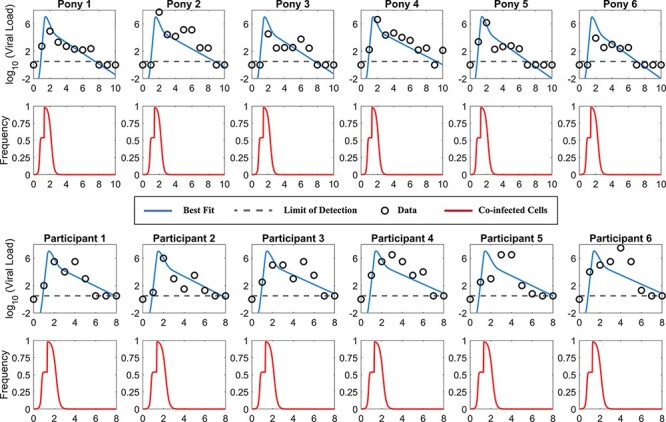
Fitting results and predictions of the TS model to two datasets, i.e. the pony experimental data from Quinlivan et al. (2007) in the upper panels and the human challenge data from Murphy et al. (1980) in the lower panels. The best-fit viral loads and predicted frequency of coinfection are shown. The data points are shown as squares, and the dotted lines denote the limit of detection of the experiment. The best-fit parameters for each individual are presented in Table 1.

Using the best-fit parameter values, we show that the model recapitulates key features of the viral load dynamics, including the exponential viral growth phase during the first 2–4 days of infection (Fig. 4). In general, we estimated that the Michaelis–Menten constant ${K_M}$ is very small (see Tables 1 and 2), indicating that the number of target cells that a virus can reach is very low. Thus, the first growth phase lasts for a very short period of time, and consequently, coinfection occurs frequently during most of the exponential periods for all individuals (Fig. 4). This suggests that SIPs alone can contribute substantially to the fully infectious viral load during the viral exponential growth.

**Table 1.** The best-fit individual estimates from fitting the TS model to two datasets using a nonlinear mixed-effect modeling approach. See Methods for further details on the fitting procedure.

ID	*K_M_* (/ml)	*δ_1_* (/day)	*δ_2_* (/day)	*p*_1_ (TCID_50_/ml/day)	*V*(0) (TCID_50_/ml)
Pony 1	30.2	1.81	10.11	5.87	6.31 × 10^−5^
Pony 2	57.5	1.52	10.12	5.36	5.01 × 10^−5^
Pony 3	46.8	1.78	10.16	5.46	5.37 × 10^−5^
Pony 4	35.5	1.42	10.10	5.74	5.89 × 10^−5^
Pony 5	28.2	1.95	10.07	5.93	6.61 × 10^−5^
Pony 6	44.7	1.97	10.17	5.93	5.37 × 10^−5^
Pt 1	33.9	1.71	10.11	5.76	6.03 × 10^−5^
Pt 2	38.0	1.77	10.11	5.63	5.75 × 10^−5^
Pt 3	32.4	1.58	10.12	5.82	6.31 × 10^−5^
Pt 4	29.5	1.55	10.10	5.93	6.46 × 10^−5^
Pt 5	30.9	1.68	10.16	5.87	6.31 × 10^−5^
Pt 6	28.2	1.56	10.11	6.01	6.61 × 10^−5^

Pt = participant.

**Table 2.** The best-fit population estimates from fitting the TS model to two datasets using a nonlinear mixed-effect modeling approach. S.E. and R.S.E. stand for standard error and relative standard error, respectively.

	Population estimate	S.E. (R.S.E.)	Random effect	S.E. (R.S.E.)
log_10_ *K_M_* (**/**ml)	1.54	4.6 (300%)	0.17	1.79 (1040%)
*δ_1_* (/day)	1.68	0.14 (8.42%)	0.14	0.38 (267%)
*δ_2_* (/day)	10.1	1.36 (13.5%)	0.075	0.15 (206%)
*P_1_* (TCID_50_**/**ml**/**day)	5.74	6.66 (116%)	0.098	0.16 (160%)
log_10_ *V_f_* (0) (/ml)	−4.22	1.22 (28.9%)	0.034	0.055 (160%)

Furthermore, although we primarily focus on the dynamics during the exponential growth period, we note that our model describes the biphasic viral decline seen after the peak viral load (Fig. 4). In our model, most infected cells are coinfected at peak viremia, and thus, the first phase of decline is driven by the death of coinfected cells. When the coinfected cells die at a faster rate than singly infected cells, over time, the coinfected cells are cleared, and singly infected cells become dominant, leading to a second phase of decline. Viral loads are mostly produced by singly infected cells during this period, and as a result, the predicted coinfection frequency and contribution of SIPs toward the viral loads dropped (see the rapid decreases after peak viremia in these individuals in Fig. 4). This is consistent with the findings of our recent study that explicitly keeping track of cell populations that are infected by different numbers of viruses (Koelle et al. 2019). We caution that this result only provides one possible explanation for the biphasic decline, among several other explanations. These other explanations instead invoke alternative processes such as the replenishment of susceptible cells through the waning of an interferon-induced refractory state (Pawelek et al. 2012) or density-dependent killing of infected cells (Smith et al. 2018). Further experiments are needed to test these hypotheses.

## Discussion

In this study, we proposed and analyzed mathematical models explicitly considering coinfection dynamics of SIPs during influenza virus infection. By comparing two alternative models against the observed data, we demonstrated the importance of incorporating the impact of spatial structure on the infection process. We showed that fully infectious particles (FIPs) from singly infected cells initially drive the spread of IAV after infection for a very short period of time, and then, coinfection becomes frequent and consequently SIPs can contribute substantially to and regulate viral load dynamics through complementation. Our model provides an explanation as to how IAV can afford to produce such a large fraction of SIPs.

Previously, influenza within-host models often use an MA term to describe the infection process by making the assumption of homogeneous mixing of cells and viruses (Baccam et al. 2006; Pawelek et al. 2012) (see Ref. Quirouette et al. (2020) for an exception). This approach ignores the spatial structure during the infection process. We showed that under this assumption, a low frequency of coinfection and a negligible contribution of SIPs toward total viral load are predicted during a large fraction of the exponential viral growth period, inconsistent with experimental observations (Jacobs et al. 2019). However, by considering the impact of spatial structure on the availability of target cells, the TS model predicts that the exponential viral growth observed in experiments can be largely driven by SIPs and coinfected cells. Viral growth rate becomes relatively insensitive to changes in the fraction of SIP production when virus production increases with increases in the multiplicity of infection (MOI). This may explain the substantial variation in SIP production between IAV strains (Brooke et al. 2013, 2014; Brooke 2017). These results strongly argue for the important role that SIPs play during infection and the crucial importance of measuring the relationship between the viral input (i.e. the MOI) and viral output of an infected cell (the consequences of MOI) (Koelle et al. 2019; Martin et al. 2020) in order to make precise predictions of viral dynamics and therapeutic interventions.

A recent experimental study showed that an engineered IAV that was unable to produce FIPs could cause productive infection when administered at a high inoculum dose to guinea pigs; however, the engineered IAV failed to be transmitted (Jacobs et al. 2019). The authors further used a cellular automaton model to show that infection can spread through the coinfection of SIPs when the spread is spatially structured. Consistent with this study, our model results suggest that at the time of initial infection, when the viral load is very low, the infection is driven by FIPs and SIPs alone are insufficient to establish infection. As viral load increases and coinfection becomes frequent, SIPs are able to contribute substantially to viral infection and spread to neighboring cells. Overall, these results demonstrate the different roles that FIPs and SIPs may play during the initial infection establishment and in regulating within-host viral dynamics once the infection is established in a host.

Reassortment is common among seasonal IAV strains, potentially contributing to an increased severity of seasonal epidemics (Holmes et al. 2005; Westgeest et al. 2014) and to the emergence of pandemic IAV strains (Taubenberger and Kash 2010). Recent work has highlighted how SIPs can enhance the frequency of IAV reassortment, a fundamental process driving IAV evolution and adaptation (Fonville et al. 2015). The prediction of our model, that coinfection is frequent *in vivo* and SIPs contribute substantially toward the total viral load, thus has important implications in understanding viral genetic diversity, reassortment, adaptation, and drug resistance development. Our model here can be a useful tool to provide an estimate of the extent of coinfection and reassortment in a host. In contrast, ignoring the contribution of SIPs to within-host viral dynamics would lead to an imprecise estimate of the active viral population size, which would in turn lead to an underestimate of the viral diversity in the within-host viral population and the probability of drug resistance (as demonstrated in Perelson, Rong, and Hayden (2012)) and the frequency of reassortment.

Despite the ability to reproduce a wealth of dynamics seen in experiments, we acknowledge that the TS model has limitations. First, our model considers the impact of the spatial spread of viral infection on the availability of target cells using a saturation function in the infection term in ODEs. A spatially explicit model (such as Huang, Dai, and Ke (2019), Quirouette et al. (2020), and Michael Lavigne et al. (2021)) may be needed to fully describe the spatial spread. However, one of the goals of our study is to compare model outputs with longitudinal viral load datasets; the high computational cost of a fully spatial model prevents us from fitting the model to data. We thus chose an implicit spatial model for analysis. Second, several recent experimental studies have shown the extreme heterogeneity in IAV infection at the single cell level (Russell, Trapnell, and Bloom 2018; Steuerman et al. 2018; Russell et al. 2019; Sun et al. 2020). This heterogeneity may play an important role in driving the initial virus stochastic infection dynamics. Our model here uses a deterministic approach and hence cannot be used to understand the stochasticity of infection arising from heterogeneous viral production. Models (such as Huang, Dai, and Ke (2019) and Michael Lavigne et al. (2021)) that incorporate both the stochastic nature of viral production and spatial spread are warranted. Another limitation is that our model assumes that cells that are infected with more than two virions are phenotypically identical to those infected with only two virions. This assumption allows us to model cells with any number of coinfecting virions within them while only explicitly modeling the coinfected cell populations (${{\rm{E}}_2}$ and ${{\rm{I}}_2}$). Modeling this system without this assumption would require either a larger, more complicated model (Dixit and Perelson 2005) or an alternate model formulation (Koelle et al. 2019).

Overall, our work provides a simple and appropriate framework to consider the impact of spatial structure and coinfection on viral dynamics. This modeling framework can be easily fit to experimental data to estimate parameters. More broadly, it is suitable and can be adapted to understand and predict the impacts of reassortment, the origins, and consequences of genetic and genomic diversity within viral populations (Brooke 2017), the roles and consequences of coinfection/superinfection during the process of drug resistance, and adaptation for influenza and other viruses (Ke et al. 2018). Furthermore, there is a growing interest in developing defective interfering particle (DIP)-based therapies (Dimmock, Easton, and Goff 2014; Kupke et al. 2019; Vignuzzi and Lopez 2019; Chaturvedi et al. 2021). Given the critical dependence of DIPs’ survival and persistence on coinfection, this model can be adapted to evaluate the efficacy of DIP-based therapies across a variety of acute viral infections.

## Methods

### The MA model

The model is described by the following system of ODEs:


$$\frac{dT}{dt}=-\beta_{MA}T{(V_F+V_S)},$$

$$\frac{{dS}}{{dt}} = {\beta _{MA}}T{V_S} - {\beta _{MA}}S\left( {{V_F} + {V_S}} \right) - kS,$$

$$\frac{{d{E_1}}}{{dt}} = {\beta _{MA}}T{V_F} - {\beta _{MA}}{E_1}\left( {{V_F} + {V_S}} \right) - k{E_1},$$

(4) $$\frac{{d{E_2}}}{{dt}} = {\beta _{MA}}\left( {S + {E_1}} \right)\left( {{V_F} + {V_S}} \right) - k{E_2},$$

$$\frac{{d{I_1}}}{{dt}} = k{E_1} - {\delta _1}{I_1},$$

$$\frac{{d{I_2}}}{{dt}} = k{E_2} - {\delta _2}{I_2},$$

$$\frac{{d{V_F}}}{{dt}} = \left( {1 - f} \right)\left( {{p_1}{I_1} + {p_2}{I_2}} \right) - c{V_F},$$

$$\frac{{d{V_S}}}{{dt}} = f\left( {{p_1}{I_1} + {p_2}{I_2}} \right) - c{V_S}.$$

In this model, target cells (*T*) are infected with FIPs (*V_F_*) and SIPs (*V_S_*) to become, respectively, FIP-infected cells (*E_1_*) and SIP-infected cells (*S*) at rate *β_MA_*. Note that viral infection events are modeled using an MA term in this model, i.e. viruses and cells are well mixed. This assumption implies that once a virion is produced, it has an equal probability to contact every cell in a host. Although this assumption is often used in models for simplicity, it is clearly biologically unrealistic, especially for influenza infection where target cells are distributed spatially from the upper to the lower respiratory tract (Gallagher et al. 2018).

FIP-infected cells go through an eclipse phase (*E_1_* cells) during which no virions are produced. *E_1_* cells then mature to singly infected virion-producing cells, *I_1_*, at rate *k*. It has been shown that a cell coinfected with two SIPs will likely induce viral production in the cell through complementation, i.e. multiplicity reactivation (Brooke 2014). Thus, we assume that if *E_1_* or *S* cells are coinfected by either a fully or semi-infectious virion, they become coinfected cells in an eclipse phase, *E_2_*, which then mature to virion-producing cells, *I_2_*, at rate *k*. For SIP-infected cells (*S*), there exists a period of time (1/*k* on average) when they can become superinfected. After this period, the cells become resistant to superinfection, and thus, these cells are removed from our system (Dou et al. 2017; Sun and BrooKe et al. 2018). Singly and coinfected virion-producing cells (*I_1_* and *I_2_*, respectively) die at per capita rate *δ_1_* and *δ_2_*, respectively. We do not consider superinfection of *I_1_* or *I_2_* cells because it has been shown that only cells in their eclipse phase are vulnerable to superinfection (Dou et al. 2017; Sun and BrooKe et al. 2018). Virions are produced from singly and coinfected virion-producing cells at rates *p_1_* and *p_2_*, respectively. Of all virions produced, a constant fraction, *f*, of them are semi-infectious (Brooke et al. 2013), while the remaining fraction are fully infectious. Both FIPs (*V_F_*) and SIPs (*V_S_*) are cleared at rate *c*. Note that to keep the model simple and because we mostly focus on the importance of incorporating cells infected by more than one virion, we do not explicitly keep track of cells infected with more than two virions. Here, we interpret the cells in the *I_2_* class as a population of cells productively infected with two or more virions. The parameters associated with *I_2_*, such as ${\delta _2}$ and ${p_2}$, then represent the average of the population.

### The TS model

The system of ODEs for the TS model is shown below.


$$\frac{{dT}}{{dt}} = - {\beta _{TS}}\frac{T}{{T + {K_M}}}\left( {{V_F} + {V_S}} \right),$$

$${{dS} \over {dt}} = {\beta _{TS}}{T \over {T + {K_M}}}{V_S} - {\beta _{TS}}{S \over {\mathbb{E} + {K_M}}}\left( {{V_F} + {V_S}} \right) - kS,$$

$${{d{E_1}} \over {dt}} = {\beta _{TS}}{T \over {T + {K_M}}}{V_F} - {\beta _{TS}}{{{E_1}} \over {\mathbb{E} + {K_M}}}\left( {{V_F} + {V_S}} \right) - k{E_1},$$

(5) $${{d{E_2}} \over {dt}} = {\beta _{TS}}{{S + {E_1}} \over {\mathbb{E} + {K_M}}}\left( {{V_F} + {V_S}} \right) - k{E_2},$$

$$\frac{{d{I_1}}}{{dt}} = k{E_1} - {\delta _1}{I_1},$$

$$\frac{{d{I_2}}}{{dt}} = k{E_2} - {\delta _2}{I_2},$$

$$\frac{{d{V_F}}}{{dt}} = \left( {1 - f} \right)\left( {{p_1}{I_1} + {p_2}{I_2}} \right) - c{V_F},$$

$$\frac{{d{V_S}}}{{dt}} = f\left( {{p_1}{I_1} + {p_2}{I_2}} \right) - c{V_S}.$$

The only differences between this model and the MA model are the Michaelis–Menten infection terms. For virus infection of uninfected cells (*T*), we use the term $\frac{T}{{T + {K_M}}}$, where *K_M_*, the saturation parameter, describes the amount of cells at which half of the maximum infectivity is reached. This type of infection term has also been used previously in human immunodeficiency virus within-host models (De Boer 2007). Here in our model, we assume that there are a limited number of target cells that can be infected (defined by *K_M_*); when the number of susceptible cells is greater than *K_M_*, the rate of infection of susceptible cells saturates to its maximum. For infection of already infected cells, i.e. *S, E_1_*, and *E_2_*, the denominator of the Michaelis–Menten term is set to E + *K_M,_* where E = *S* + *E*_1_ + *E*_2_. This is motivated by the idea that already infected cells are close to each other at an infection site (especially when the infection process is spatially structured (Matrosovich et al. 2004)), so a virion that is produced from productively infected cells can reach those eclipse phase cells in the neighborhood. Thus, we sum up all the eclipse phase cells that can be superinfected.

Note that because of the Michaelis–Menten terms we use, the units and interpretation of the parameter *β_TS_* differ from *β_MA_*. Here, *β_TS_* is the maximum infectivity rate per virion when target cells reach saturation.

### Parameter values, simulation, and data-fitting procedure

In all simulations and parameter estimations, we set the initial target cell count to *T*(0) = 4 × 10^8^ cells, based on Baccam et al. (2006), and the maturation time 1/*k* to 1/8 days (3 hours), based on Dou et al. (2017). We further set *c* = 15/day and *f* = 0.9 (dimensionless) as biologically reasonable parameter values for the virion clearance rate and the fraction of virions produced that are semi-infectious (Brooke et al. 2013; Brooke 2014), respectively.

Parameter values used to generate simulation results shown in Figs. 2 and 3 are as follows: ${\beta _{MA}}$ = 1e-5 ml/ tissue culture infectious dose (TCID_50_)/cell/day, ${\delta _1}$ = 2/day, ${\delta _2}$ = 4/day, ${p_1}$ = 1 TCID_50_/ml/day, and ${V_f}\left( 0 \right)$ = 0.01 ml/day (for Fig. 2) and ${\beta _{TS}}$ = 1,000 ml/TCID_50_/day, ${K_M}$ = 10 ml, ${\delta _1}$ = 2/day, ${\delta _2}$ = 4/day, ${p_1}$ = 1 TCID_50_/ml/day, ${V_f}\left( 0 \right)$ = 0.01 ml/day (for Fig. 3). Simulation results were generated by integrating of the MA model or the TS model using the built-in function, ode45, in MATLAB.

We took a nonlinear mixed-effect modeling approach to fit the TS model to viral load data from all individuals from the two datasets (Quinlivan et al. 2007; Pawelek et al. 2012) simultaneously. In the fitting, we fixed the values of *T*(0), *k, c*, and *f* as mentioned earlier. It is known that when fitting the viral dynamic model to viral load data, the parameters ${\beta _{TS}}$ and $p$ strongly correlated with each other, i.e. it is not possible to precisely estimate both parameter values at the same time. Thus, we fixed parameter ${\beta _{TS}}$ to 1,000 ml/TCID_50_/day (as in Fig. 3). A recent experimental study found that IAV production increases with increases in MOI, i.e. cells infected by multiple viruses produce a higher number of viral particles than cells infected by a single virus (Martin et al. 2020). Here, in our model, the coinfected cells implicitly include all cells infected by more than one virus (Dixit and Perelson 2005). Thus, we expect that the viral output from coinfected cells is much higher than that from singly infected cells, and we thus set *p_2_ *= 3*p_1_* when fitting our model to the data, roughly consistent with the experimental estimates in Martin et al. (2020).

The saturation parameter, death rates of virion-producing cells, virion production rate, and initial FIP concentration, i.e. *K_M_, δ_1_, δ_2_, p_1_*, and *V_F_*(0), were estimated from the data. We note that ${\rm{TCI}}{{\rm{D}}_{50}}$ viral titers are measurements of fully infectious virus only, and thus, we fit ${V_F}$ to the data. We calculated the initial SIP concentration, *V_S_*(0), by assuming that the viral input dose maintained the constant proportion of FIPs to SIPs seen in viral production, i.e. ${V_S}\left( 0 \right) = {V_F}\left( 0 \right)\frac{f}{{1 - f}}\,.$ While fitting the model to data, we restricted ${K_M}$ to be greater than or equal to 1 cell and assumed that *δ*_1_ ≤ *δ*_2_.

Estimations were performed using Monolix (Monolix Suite 2019R2, Antony, France: Lixoft SAS, 2019. lixoft.com/products/monolix/). All individual parameters are positive, and therefore, we assume that they follow log-normal distributions. We allowed random effects on all the fitted parameters. We tested the source of data (i.e. pony or human volunteers) as covariates on the fitted parameters and found that there is no statistically significant difference between any of the estimated parameters for the two datasets (Fig. S1).

## Supplementary Material

vead020_Supp

## Data Availability

All data are reported in the paper, and all model results of this work can be reproduced using the information provided in the manuscript.
