# Supplementary material for: Semi-infectious particles contribute substantially to influenza virus within-host dynamics when infection is dominated by spatial structure
Source: Virus Evol. 2023 Mar 21;9(1):vead020. doi: 10.1093/ve/vead020 (PMC10395763; doi:10.1093/ve/vead020)
Supplement: vead020_Supp [file vead020_supp.zip › suppl_data/2019 01 15 SIP Supplemental Material.pdf]

S1 Supplementary Text

S1.1 Analytical Approximations to the Viral Growth Rates in the Target Cell Saturation (TS) Model

S1.1.1 Outline

To derive analytical approximations to the exponential viral growth rate in the TS model, we first simplify the ODEs. Then we further simplify the system by showing that the Michaelis-Menten functions saturate and become nearly constant during each phase. Finally, we approximate the growth rates in each phase for the simplified model.

S1.1.2 The Full Model

The following system of differential equations describes the target saturation model:

$$
\begin{aligned}
\frac{dT}{dt} &= -\beta_{TS}\frac{T}{T+K_M}(V_F+V_S),\\
\frac{dV_F}{dt} &= (1-f)(p_1I_1+p_2I_2)-cV_F,\\
\frac{dV_S}{dt} &= f(p_1I_1+p_2I_2)-cV_S,\\
\frac{dS}{dt} &= \beta_{TS}\frac{T}{T+K_M}V_S-\beta_{TS}\frac{S}{\sum\mathbb{E}+K_M}(V_F+V_S)-kS,\\
\frac{dE_1}{dt} &= \beta_{TS}\frac{T}{T+K_M}V_F-\beta_{TS}\frac{E_1}{\sum\mathbb{E}+K_M}(V_F+V_S)-kE_1,\\
\frac{dE_2}{dt} &= \beta_{TS}\frac{S+E_1}{\sum\mathbb{E}+K_M}(V_F+V_S)-kE_2,\\
\frac{dI_1}{dt} &= kE_1-\delta I_1,\\
\frac{dI_2}{dt} &= kE_2-\delta I_2,
\end{aligned}
\tag{1}
$$

with $\sum\mathbb{E}=S+E_1+E_2$.

We assume that $V_F(0)=\frac{1-f}{f}V_S(0)$ and that all infected cell populations have zero population initially.

We further assume that $K_M << T(0)$. If this is not the case, the Michaelis-Menten terms can be approximated by a mass action terms, as seen in Section S1.2.3.

S1.1.3 Model Reduction

Mathematical Preliminary: Showing That Solutions Are Bounded and Lipschitz Continuous

Define $N := T + S + E_1 + E_2 + I_1 + I_2$ as the total cell population. We have assumed that there are only target cells and virions present initially, so $N(0) = T(0)$. We see that

$$\frac{dN}{dt} = -\delta I_1 - \delta I_2 - kS,$$

which implies $N(t) \leq N(0) = T(0)$. Thus, the total cell population is bounded by $T(0)$, implying that each individual cell population is bounded by it as well. Since I_1 and I_2 are bounded, the differential equations for V_F and V_S imply that the viral populations will be bounded as well.

Thus, all of our variables are bounded and so are their derivatives. Notably, this implies that the variables are Lipschitz continuous, which provides uniqueness of solutions to the system.

The Relationship between $\boldsymbol{V_F}$ and $\boldsymbol{V_S}$

We see that

$$\frac{dV_F}{dt} = (1-f)(p_1 I_1 + p_2 I_2) - cV_F$$

and

$$\frac{d}{dt}\Big[\frac{1-f}{f}V_S\Big] = (1-f)(p_1 I_1 + p_2 I_2) - c\Big(\frac{1-f}{f}V_S\Big)$$

both have the same form. Thus, since they are both Lipschitz continuous and $\frac{1-f}{f}V_S(0) = V_F(0)$ (assumed in Section S1.1.2), we have $\frac{1-f}{f}V_S(t) = V_F(t)$. This result follows from the uniqueness of ODEs.

We define $V := \frac{V_F}{1-f} = \frac{V_S}{f}$, which implies $V = V_F + V_S$. Thus V is the total viral load.

This allows us to make the following replacements:

$$\begin{aligned} V_S(t) &= fV(t), \\ V_F(t) &= (1-f)V(t), \\ V_F(t) + V_S(t) &= V(t). \end{aligned} \tag{2}$$

The Relationship between $\boldsymbol{S}$ and $\boldsymbol{E_1}$

In a similar manner as above, we have the following

$$\begin{aligned} S(t) &= fE_T(t) \\ E_1(t) &= (1-f)E_T(t) \\ E_T(t) &:= E_1(t) + S(t). \end{aligned} \tag{3}$$

The Reduced Model

Using Eqns. (2) and (3) we can reduce Eqns. (1) to the following:

$$\begin{aligned} \frac{dT}{dt} &= -\beta_{TS} C_\tau(t) V, \\ \frac{dV}{dt} &= (p_1 I_1 + p_2 I_2) - cV, \\ \frac{dE_T}{dt} &= \beta_{TS} C_\tau(t) V - \beta_{TS} C_\gamma(t) V - kE_T, \\ \frac{dE_2}{dt} &= \beta_{TS} C_\gamma(t) V - kE_2, \\ \frac{dI_1}{dt} &= k(1-f)E_T - \delta I_1, \\ \frac{dI_2}{dt} &= kE_2 - \delta I_2, \end{aligned} \tag{4}$$

with $C_\tau(t) := \dfrac{T(t)}{T(t) + K_M}$, $C_\gamma(t) := \dfrac{E_T(t)}{\sum \mathbb{E}(t) + K_M}$, and $\sum \mathbb{E}(t) = E_T(t) + E_2(t)$.

Approximating the Michaelis-Menten Functions

We hypothesize that the Michaelis-Menten functions $C_\tau(t)$ and $C_\gamma(t)$ will saturate to be approximately constant during each phase of growth.

We now determine the piecewise approximations of C_τ and C_γ during each of the three phases:

- First phase, I_1 driven growth:

$$C_\tau \approx 1, \quad \text{and} \quad C_\gamma \approx 0.$$

  Coinfection is negligible. Occurs if $\sum \mathbb{E} << K_M << T$.

- Second phase, I_2 driven growth:

$$C_\tau \approx 1, \quad \text{and} \quad C_\gamma \approx \gamma,$$

  where $\gamma \in (0,1)$. Coinfection levels have reached their maximum. The coinfection related Michaelis-Menten term has saturated and is essentially constant. Occurs if $K_M << E_T < \sum \mathbb{E}$ and $K_M << T$.

- Third phase, target cell depletion and viral decline:

$$C_\tau \approx 0.$$

  Looking at the differential equation for E_T, we see that this causes an extremely rapid decline of E_T, leading to

$$C_\gamma \approx 0.$$

  Occurs if $T << K_M$.

Thus, we have the following piecewise approximations for C_τ and C_γ during each phase:

$$\begin{aligned} C_\tau &\approx \begin{cases} 1 & \text{Phases 1 \& 2} \\ 0 & \text{Phase 3,} \end{cases} \\ C_\gamma &\approx \begin{cases} 0 & \text{Phases 1 \& 3} \\ \gamma & \text{Phase 2.} \end{cases} \end{aligned} \tag{5}$$

We note that these approximations do not hold during transitional periods between phases.

Calculating the Value of γ

In this section we assume that we are in the second phase of growth, allowing us to use the values from Eqn. (5) to approximate C_τ and C_γ. To calculate

γ we only need the differential equations for E_T, and E_2 from Eqns. (4), yielding

$$\frac{dE_T}{dt} = \beta_{TS}V - \beta_{TS}\gamma V - kE_T$$
$$\frac{dE_2}{dt} = \beta_{TS}\gamma V - kE_2.$$

Based on simulations, we assume that the virus can be approximated by exponential growth: $V(t) \approx V_0 e^{\psi t}$, for some $\psi > 0$ during this phase. With this, the differential equations are solvable. For E_T, we have $\frac{dE_T}{dt} \approx \beta(1 - \gamma)V_0 e^{\psi t} - kE_T$, which has solution $E_T(t) \approx \frac{V_0\beta(1-\gamma)}{\psi + k}\left(e^{\psi t} - e^{-kt}\right)$. Taking the largest exponent, we can approximate E_T with

$$E_T(t) \approx \frac{V_0\beta(1-\gamma)}{\psi + k} e^{\psi t}.$$

Similarly we find

$$E_2(t) \approx \frac{V_0\beta_{TS}\gamma}{\psi + k} e^{\psi t}.$$

With these approximate solutions, we can calculate $\sum \mathbb{E}$.

$$\sum \mathbb{E} = E_T + E_2 \approx \frac{\beta_{TS}V_0}{\psi + k} e^{\psi t}.$$

Since we assume that we are in the second phase of growth we can also make the approximation $\sum \mathbb{E} + K_M \approx \sum \mathbb{E}$.

$$\gamma = \frac{E_T}{\sum \mathbb{E} + K_M} \approx \frac{E_T}{\sum \mathbb{E}} = \frac{\frac{\beta_{TS}V_0(1-\gamma)}{\psi + k} e^{\psi t}}{\frac{\beta_{TS}V_0}{\psi + k} e^{\psi t}} = 1 - \gamma,$$

which yields $\gamma = \frac{1}{2}$.

This allows us to update Eqn. (5):

$$C_\tau \approx \begin{cases} 1 & \text{Phases 1 \& 2} \\ 0 & \text{Phase 3,} \end{cases}$$
$$C_\gamma \approx \begin{cases} 0 & \text{Phases 1 \& 3} \\ \frac{1}{2} & \text{Phase 2.} \end{cases} \tag{6}$$

S1.1.4 Estimates of Viral Growth Rate in Each Phase

Beginning with Eqns. (4), we define $E := p_1(1-f)E_T + p_2E_2$ and $I := p_1I_1 + p_2I_2$, giving the following ODE system:

$$\begin{aligned}
\frac{dT}{dt} &= -\beta_{TS}C_\tau(t)V \\
\frac{dV}{dt} &= I - cV \\
\frac{dE}{dt} &= \beta_{TS}(p_1(1-f)(C_\tau(t) - C_\gamma(t)) + p_2C_\gamma(t))V - kE \\
\frac{dI}{dt} &= kE - \delta I
\end{aligned}$$

For ease of writing, we define the composite parameter

$$\alpha = \beta_{TS}\Big(p_1(1-f)\big(C_\tau(t) - C_\gamma(t)\big) + p_2C_\gamma(t)\Big). \tag{7}$$

Using Eqns. (6), we can write a piecewise definition of α in each phase.

$$\alpha = \begin{cases} \beta_{TS}p_1(1-f) & \text{Phase 1,} \\ \beta_{TS}\Big(\dfrac{p_1}{2}(1-f) + \dfrac{p_2}{2}\Big) & \text{Phase 2,} \\ 0 & \text{Phase 3.} \end{cases}$$

We note that we can rewrite α in phase 2 as $\beta_{TS}p_1\big(1 - \frac{f}{2} + \frac{p_2-p_1}{2p_1}\big)$. Further, we see that V, E, and I form a closed system:

$$\begin{aligned}
\frac{dV}{dt} &= I - cV \\
\frac{dE}{dt} &= \alpha V - kE \\
\frac{dI}{dt} &= kE - \delta I.
\end{aligned} \tag{8}$$

While analysis can be done on the model in this form, the eigenvalues will be given as roots of a third degree polynomial, whose analytical expressions are too long to be useful. To find a more tractable form that will still provide an intuition for how SIPs affect viral growth we make a quasi stead state assumption on the viral compartment, reducing the system to two dimensions.

$$\frac{dE}{dt} = \frac{\alpha}{c}I - kE,$$
$$\frac{dI}{dt} = kE - \delta I.$$

This system has eigenvalues

$$\lambda_{+,-} = \frac{-\delta - k \pm \sqrt{(\delta + k)^2 + 4k\delta\Big(\frac{\alpha}{c\delta} - 1\Big)}}{2}. \tag{9}$$

Note that this quantity will be positive if and only if $1 < \frac{\alpha}{c\delta}$.

The larger root will determine the overall, non-transient growth rate, so we use the positive branch of $\lambda_{+,-}$ and remove the subscript.

We now show λ in each phase after substituting in the appropriate approximation for α. In phase 1, we have

$$\lambda_1 \approx \frac{-\delta - k + \sqrt{(\delta + k)^2 + 4k\delta\Big(\frac{\beta_{TS}p_1}{c\delta}(1 - f) - 1\Big)}}{2}$$

Note that in the deravation above, we assumed the death rates of cells infected by a FIP and cells co-infected by FIPs and/or SIPs are the same, i.e. δ. Since phase 1 of the exponential growth is mostly driven by cells infected by a FIP, i.e. the co-infected cells does not meaningfuly participate in the early dynamics, δ can be replaced by δ_1, and thus,

$$\lambda_1 \approx \frac{-\delta_1 - k + \sqrt{(\delta_1 + k)^2 + 4k\delta_1\Big(\frac{\beta_{TS}p_1}{c\delta_1}(1 - f) - 1\Big)}}{2} \tag{10}$$

Similarly, in phase 2, δ can be approximated by δ_2. We get

$$\lambda_2 \approx \frac{-\delta_2 - k + \sqrt{(\delta_2 + k)^2 + 4k\delta_2\Big(\frac{\beta_{TS}p_1}{c\delta_2}\Big[\frac{1-f}{2} + \frac{p_2}{2p_1}\Big] - 1\Big)}}{2}. \tag{11}$$

In the special case when $p_1 = p_2$, we can rewrite λ_2 to be

$$\lambda_2 \approx \frac{-\delta_2 - k + \sqrt{(\delta_2 + k)^2 + 4k\delta_2\Big(\frac{\beta_{TS}p_1}{c\delta_2}\Big[1 - \frac{f}{2}\Big] - 1\Big)}}{2}.$$

S1.2 Viral Growth Rates in the Mass-Action (MA) Model

S1.2.1 The Model

Rewriting Eqns. (1) using mass action kinetics yields the homogeneous mixing model:

$$\begin{aligned}
\frac{dT}{dt} &= -\beta_{MA}T(V_F + V_S),\\
\frac{dV_F}{dt} &= (1-f)(p_1 I_1 + p_2 I_2) - cV_F,\\
\frac{dV_S}{dt} &= f(p_1 I_1 + p_2 I_2) - cV_S,\\
\frac{dS}{dt} &= \beta_{MA}TV_S - \beta_{MA}S(V_F + V_S) - kS,\\
\frac{dE_1}{dt} &= \beta_{MA}TV_F - \beta_{MA}E_1(V_F + V_S) - kE_1,\\
\frac{dE_2}{dt} &= \beta_{MA}(S + E_1)(V_F + V_S) - kE_2,\\
\frac{dI_1}{dt} &= kE_1 - \delta I_1,\\
\frac{dI_2}{dt} &= kE_2 - \delta I_2,
\end{aligned} \tag{12}$$

S1.2.2 Approximation of the Initial Viral Growth Rate

First, we note that the assumptions required to create Eqn. (2) are still met by the MA model, and thus it is still valid. We further note that for a majority of the infection, $T >> E_1, T >> S$, and $I_1 >> I_2$. Finally, we suppose that $T(t) \approx T(0)$ during viral growth. This allows us to make the following approximations:

$$\begin{aligned}
\frac{dV}{dt} &\approx p_1 I_1 - cV,\\
\frac{dE_1}{dt} &\approx \beta_{MA}(1-f)T(0)V - kE_1,\\
\frac{dI_1}{dt} &\approx kE_1 - \delta I_1.
\end{aligned}$$

This has the same form as Eqn. (8), and so we can follow the calculations previously used to see that for the MA model,

$$\lambda_{MA} \approx \frac{-\delta_1 - k + \sqrt{(\delta_1 + k)^2 + 4k\delta_1\Big(\frac{\beta_{MA}T(0)p_1}{c\delta_1}(1-f) - 1\Big)}}{2}. \tag{13}$$

S1.2.3 Mass Action Kinetics can be Approximated by Michaelis-Mention Kinetics for Large K_M

The Michaelis-Menten infection term for target cells, SIP eclipse phase cells, and FIP eclipse cells are

$$\beta_{TS}\frac{T}{T+K_M}(V_F+V_S), \qquad \beta_{TS}\frac{S}{\sum \mathbb{E}+K_M}(V_F+V_S), \qquad \beta_{TS}\frac{E_1}{\sum \mathbb{E}+K_M}(V_F+V_S).$$

By the calculations in Section S1.1.3, we know that $T(t), S(t), E_1(t) \leq T(0)$ for all $t \geq 0$. Therefore, $K_M >> T(0)$ implies $K_M >> T(t)$ and $K_M >> \sum \mathbb{E}(t)$ for all $t \geq 0$. This allows us to make the following approximations: $T + K_M \approx K_M$ and $\sum \mathbb{E} + K_M \approx K_M$. Define $\phi = \frac{\beta_{TS}}{K_M}$. Then, we see

$$\beta_{TS}\frac{T}{T+K_M}(V_F+V_S) \approx \phi T(V_F+V_S), \qquad \beta_{TS}\frac{S}{\sum \mathbb{E}+K_M}(V_F+V_S) \approx \phi S(V_F+V_S),$$

$$\text{and} \qquad \beta_{TS}\frac{E_1}{\sum \mathbb{E}+K_M}(V_F+V_S) \approx \phi E_1(V_F+V_S).$$

Thus, for large K_M, Michaelis-Menten kinetics can be approximated by mass action kinetics.
