# Supplementary material for: Semi-infectious particles contribute substantially to influenza virus within-host dynamics when infection is dominated by spatial structure
Source: Virus Evol. 2023 Mar 21;9(1):vead020. doi: 10.1093/ve/vead020 (PMC10395763; doi:10.1093/ve/vead020)
Supplement: vead020_Supp [file vead020_supp.zip › suppl_data/SIPs_ms_20230217_suppl.pdf]

Supporting Figures and Table for

'Semi-infectious particles contribute substantially to influenza virus within-host dynamics when infection is dominated by spatial structure'

Alex Farrell[1], Tin Phan[2], Christopher Brooke[3, 4], Katia Koelle[5], Ruian Ke[2, *]

[1] Department of Mathematics, University of Arizona, Tucson, AZ

[2] T-6, Theoretical Biology and Biophysics, Los Alamos National Laboratory, Los Alamos, NM 87545

[3] Department of Microbiology, University of Illinois at Urbana-Champaign, IL 61801

[4] Carl R. Woese Institute for Genomic Biology, University of Illinois at Urbana-Champaign, IL 61801

[5] Department of Biology, Emory University, Atlanta, GA30322

[*] Corresponding author: rke@lanl.gov

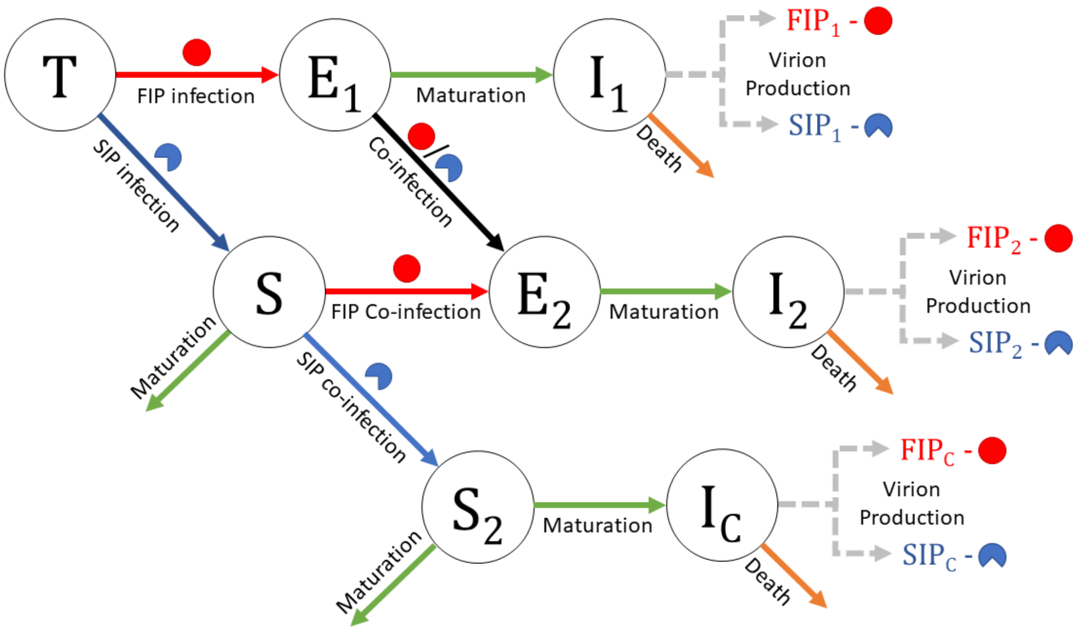

Fig. S1. Schematic of expanded model used to track virions created from multiplicity reactivation. S_2 cells have been infected with two SIPs. They then mature to I_C, which are virion producing cells derived from complementation (or multiplicity reactivation). In this formulation, E_1 cells are cells that contain both a FIP and a SIP, which mature to I_2 cells that produce virions. Thus, we can determine the fraction of virions that are due to complementation by the following equation: $\frac{FIP_C}{FIP_1+FIP_2+FIP_C}$. In this formulation, the proportion of co-infected cells is given by $\frac{E_2+I_2+S_2+I_C}{S+E_1+E_2+I_1+I_2+S_2+I_C}$ (note that this quantity is the same as $\frac{E_2+I_2}{S+E_1+E_2+I_1+I_2}$ in the original schematic in Fig. 1).

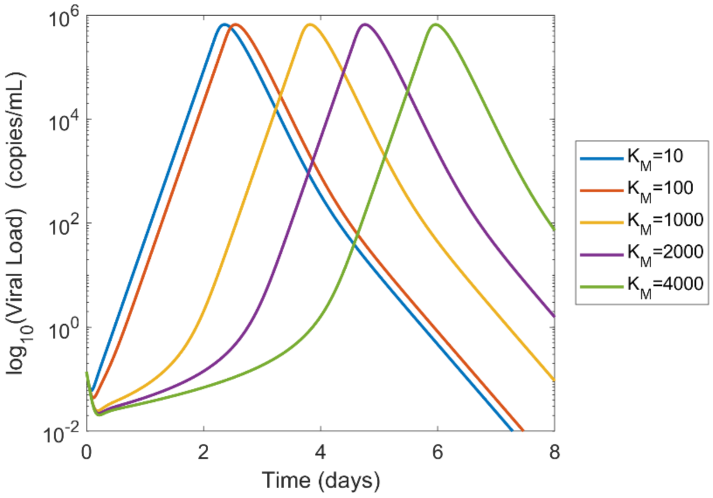

Fig. S2. Simulations showing how K_M affects the length of the first phase of growth. We see that increasing the value of K_M increases the length of the first phase of growth.

Table S1. Comparison of model results when assuming a covariate (for the two different datasets) on different sets of fitted parameters in the non-linear mixed effect modeling.

Covariates	-2LL	BICc
$K_M, \delta_1, \delta_2, p, V_f(0)$	342.9	396.4
$\delta_1, \delta_2, V_f(0)$	342.7	391.3
$\delta_1, V_f(0)$	342.2	388.3
None	346.8	387.9
